# Research hotspots and trends of brain-computer interface technology in stroke: a bibliometric study and visualization analysis

**DOI:** 10.3389/fnins.2023.1243151

**Published:** 2023-08-31

**Authors:** Fangcun Li, Ding Zhang, Jie Chen, Ke Tang, Xiaomei Li, Zhaomeng Hou

**Affiliations:** ^1^Department of Rehabilitation Medicine, Guilin Municipal Hospital of Traditional Chinese Medicine, Guilin, China; ^2^Graduate School, Guangxi University of Chinese Medicine, Nanning, China; ^3^Department of Pharmacy, Guilin Municipal Hospital of Traditional Chinese Medicine, Guilin, China; ^4^Department of Orthopedics and Traumatology, Yancheng TCM Hospital Affiliated to Nanjing University of Chinese Medicine, Yancheng, China; ^5^Department of Orthopedics and Traumatology, Yancheng TCM Hospital, Yancheng, China

**Keywords:** brain-computer interface, stroke, bibliometric, visualization analysis, VOSviewer, CiteSpace

## Abstract

**Background:**

The incidence and mortality rates of stroke are escalating due to the growing aging population, which presents a significant hazard to human health. In the realm of stroke, brain-computer interface (BCI) technology has gained considerable attention as a means to enhance treatment efficacy and improve quality of life. Consequently, a bibliometric visualization analysis was performed to investigate the research hotspots and trends of BCI technology in stroke, with the objective of furnishing reference and guidance for future research.

**Methods:**

This study utilized the Science Citation Index Expanded (SCI-Expanded) within the Web of Science Core Collection (WoSCC) database as the data source, selecting relevant literature published between 2013 and 2022 as research sample. Through the application of VOSviewer 1.6.19 and CiteSpace 6.2.R2 visualization analysis software, as well as the bibliometric online analysis platform, the scientific knowledge maps were constructed and subjected to visualization display, and statistical analysis.

**Results:**

This study encompasses a total of 693 relevant literature, which were published by 2,556 scholars from 975 institutions across 53 countries/regions and have been collected by 185 journals. In the past decade, BCI technology in stroke research has exhibited an upward trend in both annual publications and citations. China and the United States are high productivity countries, while the University of Tubingen stands out as the most contributing institution. Birbaumer N and Pfurtscheller G are the authors with the highest publication and citation frequency in this field, respectively. *Frontiers in Neuroscience* has published the most literature, while *Journal of Neural Engineering* has the highest citation frequency. The research hotspots in this field cover keywords such as stroke, BCI, rehabilitation, motor imagery (MI), motor recovery, electroencephalogram (EEG), neurorehabilitation, neural plasticity, task analysis, functional electrical stimulation (FES), motor impairment, feature extraction, and induced movement therapy, which to a certain extent reflect the development trend and frontier research direction of this field.

**Conclusion:**

This study comprehensively and visually presents the extensive and in-depth literature resources of BCI technology in stroke research in the form of knowledge maps, which facilitates scholars to gain a more convenient understanding of the development and prospects in this field, thereby promoting further research work.

## Introduction

Stroke is a common cerebrovascular disease characterized by high disability and mortality rates (Soekadar et al., 2015; Cao et al., 2022b; Zhan et al., 2022; Lin et al., 2023). With the aging of the population and changes in lifestyle, the incidence of stroke has been increasing year by year, making it one of the major challenges to global health (Chen X. et al., 2022; Zhang R. et al., 2023). Stroke patients often require long-term treatment and rehabilitation training (Cao et al., 2022a; Uivarosan et al., 2022; Liu X. et al., 2023). Therefore, improving the treatment effectiveness and rehabilitation quality has become an urgent problem in the field of stroke treatment. With advances in computer technology and neuroscience, brain-computer interface (BCI) technology has been receiving more and more attention and research in the treatment and rehabilitation of stroke patients (Mane et al., 2020; Liu et al., 2022). BCI technology is an emerging technology that connects the human brain with external devices, using the neural activity of the human brain to control external devices for interaction and control, thereby giving patients a sense of autonomous control and accelerating the rehabilitation process (Gutierrez-Martinez et al., 2021; Gao et al., 2022). Supported by BCI technology, nerve regeneration and functional recovery can be promoted, improving the quality of life and alleviating the impact caused by the disease (Sinha et al., 2021; Song et al., 2022). Hence, BCI technology has significant value and potential in improving the quality of life for stroke patients and has become one of the research hotspots in related academic fields both domestically and internationally (Hu et al., 2021; Li H. et al., 2023). In recent years, the application of BCI technology in the stroke field has gained popularity, and consequently, the number of academic literatures on this subject has been continuously increasing. Therefore, in order to systematically, objectively, and comprehensively understand the current development, research focal points, and trends of this field, the method of bibliometrics has been introduced into the research of the field, helping us to quickly analyze the quantitative characteristics and laws of these literature, in order to provide direction and reference for future research. Bibliometrics is a discipline that employs statistical and quantitative methods to study, evaluate, and analyze scientific literature (Chen Y. et al., 2022). It has been widely applied in various research fields (Luo and Lin, 2021; Zhang L. et al., 2022, 2023; Zhang and Lin, 2023).

This study systematically and comprehensively sorted and analyzed relevant literature on the use of BCI in stroke research fields using bibliometrics and visualization technologies. Firstly, this paper statistically analyzes the relevant literature on multiple aspects including total amount, annual publication, citation frequency, research countries, institutions, authors, and journals, in order to achieve a comprehensive description of the research situation in this field. Secondly, based on the scientometrics method, this paper conducts an in-depth analysis of the research hotspots, topics, and trends of the literature. On this basis, the development prospects and future research directions of BCI technology in stroke treatment are discussed. Finally, visualization techniques are adopted to present the results of literature analysis through forms such as tables and knowledge maps, which improves readability and intuitiveness, and better conveys relevant research information. Through these analyses and presentations, this research aims to unveil the current research status, prominent areas of investigation, and emerging trends of BCI technology in stroke treatment and rehabilitation, in order to furnish unique insights and directional guidance for further research and practice in this field.

## Materials and methods

### Data source and search strategy

The data source of this study was the Science Citation Index Expanded (SCI-Expanded) within the Web of Science Core Collection (WoSCC) database. To avoid potential bias caused by database updates, the literature search and data extraction were carried out on the same day. To enhance the accuracy of the search, we obtained subject headings from the Medical Subject Headings (MeSH) and constructed the search strategy using a combination of subject terms and free terms. The search formula was set as ((((TS = (“brain-computer interface” OR “brain-computer interfaces” OR “brain-machine interface” OR “brain-machine interfaces”)) AND TS = (stroke OR apoplexy OR “cerebrovascular accident” OR “cerebrovascular accidents” OR “brain vascular accident” OR “brain vascular accidents”)) AND DT = (Article OR Review)) AND LA = (English)) AND DOP = (2013-01-01/2022-12-31). After screening, proceeding papers (8), book chapters (6), and early access (3) were excluded, leaving a final total of 693 relevant publications that met the inclusion criteria.

### Bibliometric analysis

The relevant literature meeting the inclusion criteria was exported as a plain text file of “full record and cited references” and named “download_xxx.txt.” The exported literature was then imported into VOSviewer 1.6.19 and CiteSpace 6.2.R2 software for the purpose of constructing knowledge maps and conducting statistical analysis. Meanwhile, the literature was also exported in a tab delimited file and uploaded to an online analysis platform of document metrology^1^ to generate a national/regional cooperation network knowledge map. The VOSviewer software was utilized with the following parameter settings: association strength was chosen as the normalization method, and the minimum thresholds for country/region, institution, and author were established at 5, 6, and 8 respectively, based on the number of publications. Meanwhile, the minimum threshold of 80, 150, and 50 citations, respectively, was established for authors, journals, and literature. Additionally, the frequency of occurrence for keywords was also taken into account, with a minimum threshold of 20. The CiteSpace software was configured with the following parameter settings: the time span ranging from January 2013 to December 2022, with a time slice of 1 year. Node types were set to include keyword and reference, and the selection criteria were established to identify the top 50 for each slice. Pruning options, including pathfinder, sliced networks pruning, and merged network pruning, were employed, while all other settings were retained as default.

## Results

### Analysis of annual publications and citations

**Figure 1:**
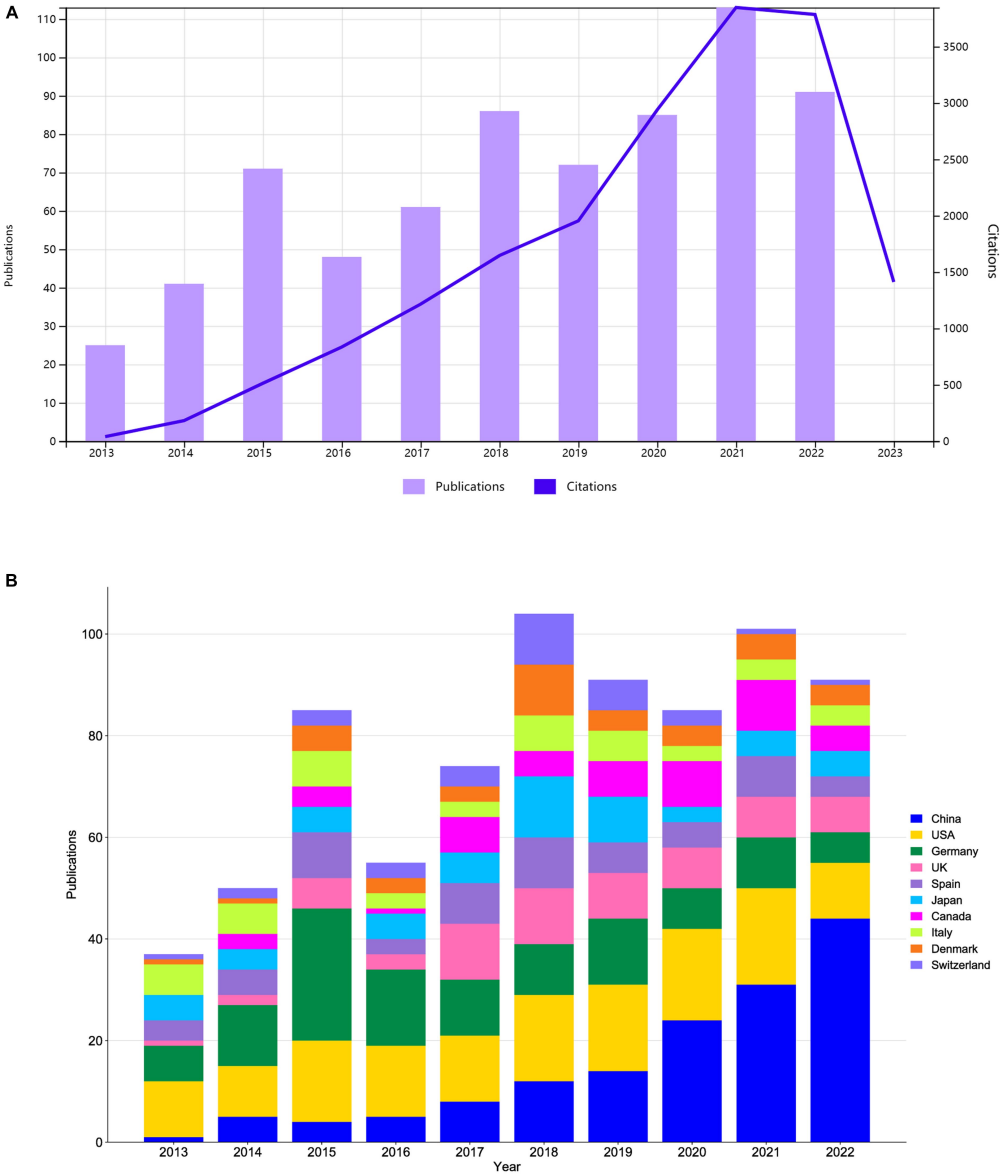
**(A)** Annual publications and citations trend chart. **(B)** Stack bar plot of top 10 countries/regions in publications.

A total of 693 relevant literatures were included in this study, of which 596 were original research articles, accounting for 86%, and 97 were review papers, accounting for 14%. The total citation frequency was 18,367, with an average citation frequency of 26.5 per paper, and an H-index of 64. As shown in Figure 1A, BCI technology in stroke research has generally shown a fluctuating upward trend in terms of annual publications and citation frequency over the past decade. The number of publications increased almost four-fold, from 25 in 2013 to 91 in 2022, while the citation frequency increased almost one hundred-fold, from 39 in 2013 to 3,785 in 2022. These results indicate sustained and widespread scholarly attention to this research field, particularly in the last 3 years when a rapid increase in the citation frequency was observed, reflecting a sustained high level of research interest. Figure 1B illustrates the annual trend in publications for the 10 countries/regions with the highest number of published works. It can be seen that China, the United States, and Germany remain the major research nations in this field. From 2013 to 2017, China had relatively few publications, with an annual average of less than five articles. However, in the past 5 years, China’s publication output has grown rapidly, surpassing Germany in 2018 to become the second highest producing country in terms of annual publications. Since 2020, China has successfully overtaken the United States to become the top producing country in terms of annual publications. This indicates that although China started later in this research field, it has since made significant strides and achieved impressive levels of productivity in the academic realm.

### Analysis of countries/regions

**Figure 2:**
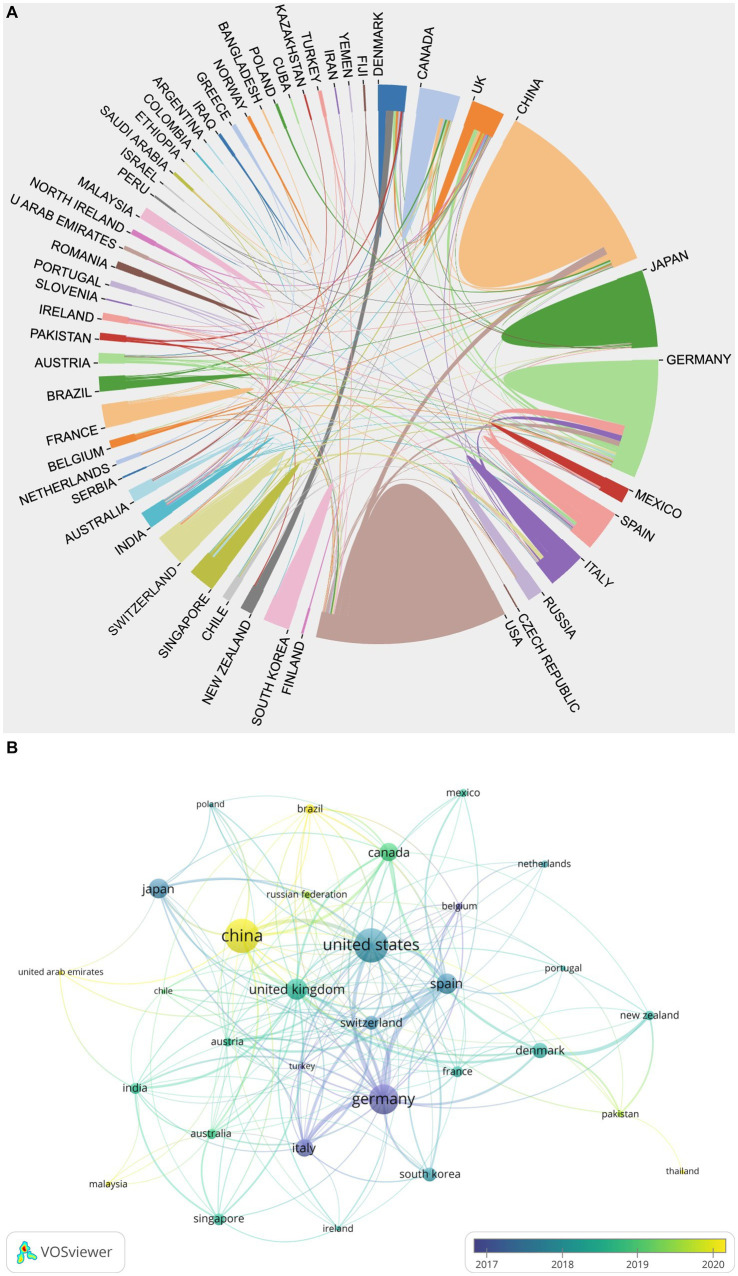
**(A)** National/regional collaborative network knowledge map. **(B)** Temporal map of national/regional cooperation network.

According to statistical analysis, a total of 53 countries/regions have participated in relevant research in the field. The national/regional cooperation network knowledge map (Figure 2A) illustrates the cooperation situation among various countries/regions, with different color blocks representing different countries/regions, and the size of the color blocks indicating the number of published articles. The lines connecting the color blocks represent the level of cooperation, with the thickness of the lines indicating the strength of the relationship (Hou et al., 2022). In addition, Figure 2B shows the temporal overlay of national/regional cooperation network, with each node representing a country/region, and the lines between nodes indicating the existence of a cooperation relationship. The thickness of the lines reflects the degree of collaboration intensity, while the color of the nodes toward yellow reflects a later average publication time (Xu et al., 2023; Yang Z. et al., 2023; Zhao et al., 2023). Therefore, it can be inferred that China (148, accounting for 21.36%) is the most active country, followed by the United States (146, accounting for 21.07%) and Germany (118, accounting for 17.03%). The sum of the number of published articles from these three countries accounts for 59% of the total publications and represents the main contributors in the field. The United States has the highest total citations (TC) and H-index, while Germany and Switzerland are leading in terms of total link strength (TLS) and average citation per publication (ACPP), respectively, as shown in Table 1. The H-index is commonly used to measure academic influence, while TLS reflects the strength of mutual collaboration (Lu et al., 2022; Huang et al., 2023; Zhang J.-Y. et al., 2023).

**Table 1:** Top 10 countries/regions ranked by number of publications.

Rank	Countries/regions	Counts (%)	TC	ACPP	H-index	TLS
1	China	148 (21.36%)	2,258	15.26	27	76
2	United States	146 (21.07%)	5,034	34.48	39	110
3	Germany	118 (17.03%)	4,984	42.24	38	162
4	United Kingdom	66 (9.52%)	1,580	23.94	24	100
5	Spain	62 (8.95%)	2,517	40.60	27	105
6	Japan	59 (8.51%)	1,628	27.59	22	31
7	Canada	51 (7.36%)	914	17.92	20	48
8	Italy	49 (7.07%)	2,630	53.67	26	72
9	Denmark	40 (5.77%)	1,140	28.50	15	57
10	Switzerland	34 (4.91%)	1,923	56.56	22	56

TC, total citations; ACPP, average citation per publication; TLS, Total link strength.

### Analysis of institutions

**Figure 3:**
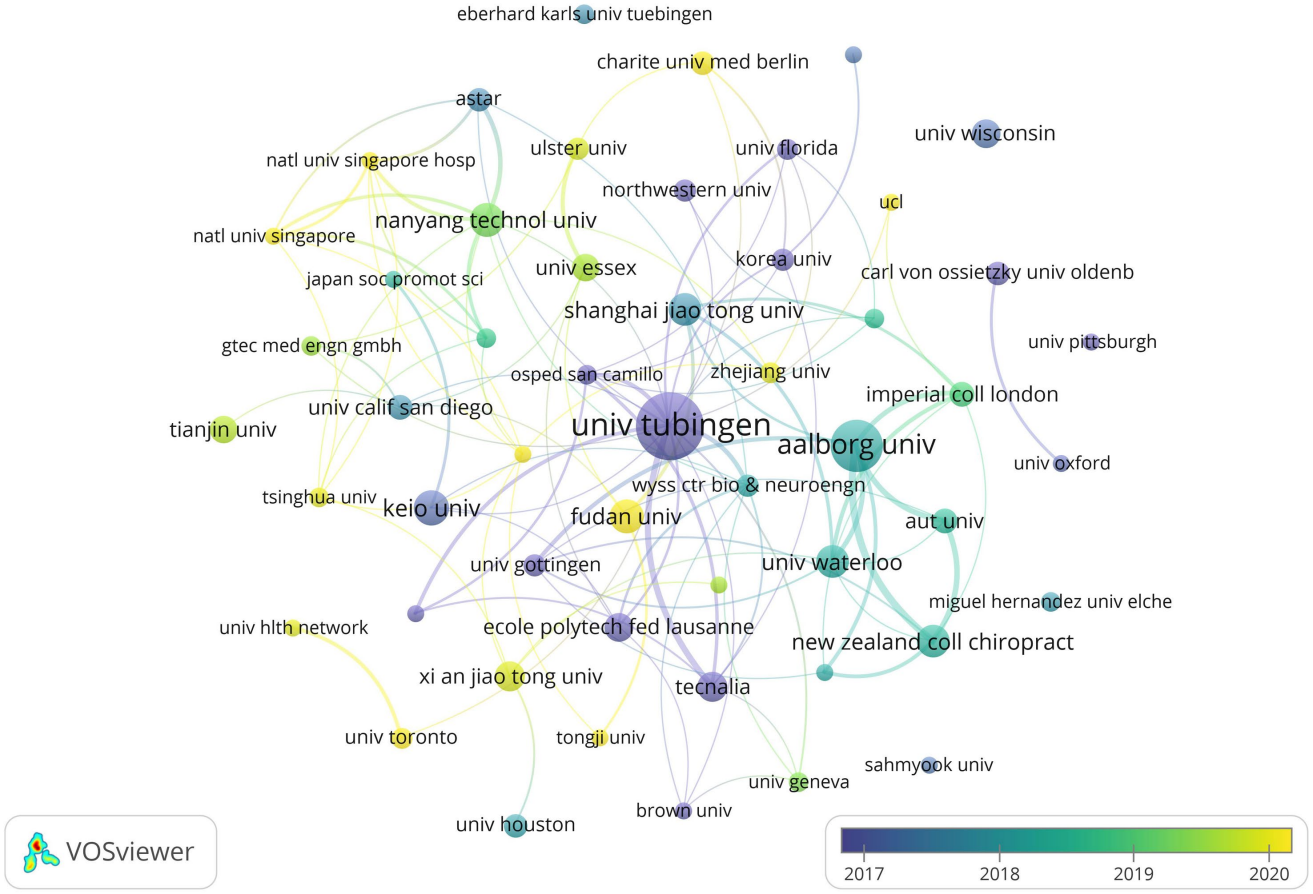
Temporal map of institutional collaborative network.

Based on statistical data, a total of 975 research institutions collaborated to publish 693 publications, and the collaboration network visualization of institutions with no less than 6 publications is shown in Figure 3. Notably, The University of Tubingen in Germany demonstrated the most outstanding performance, contributing 68 papers, accounting for 9.81% of the total publications, followed by Aalborg University (35, 5.05%) in Denmark and Fudan University (20, 2.89%) in China. It is worth mentioning that the University of Tubingen also boasted the highest TC and H-index among the institutions, while Aalborg University and Tecnalia in Spain were the institutions with the highest TLS and ACPP, respectively. Please refer to Table 2 for further details.

**Table 2:** Top 10 institutions ranked by number of publications.

Rank	Institutions	Counts (%)	TC	ACPP	H-index	TLS	Location
1	University of Tubingen	68 (9.81%)	3,306	48.62	30	55	Germany
2	Aalborg University	35 (5.05%)	890	25.43	14	62	Denmark
3	Fudan University	20 (2.89%)	342	17.10	9	10	China
4	Keio University	19 (2.74%)	676	35.58	11	7	Japan
5	Nanyang Technological University	19 (2.74%)	599	31.53	10	29	Singapore
6	New Zealand Coll Chiropract	17 (2.45%)	308	18.12	10	38	New Zealand
7	Shanghai Jiao Tong University	17 (2.45%)	460	27.06	13	17	China
8	University of Waterloo	16 (2.31%)	270	16.88	11	26	Canada
9	University of Wisconsin	15 (2.17%)	387	25.80	11	0	USA
10	Tecnalia	14 (2.02%)	1,255	89.64	11	24	Spain
11	Xi an Jiaotong University	14 (2.02%)	240	17.14	8	6	China

### Analysis of authors

**Figure 4:**
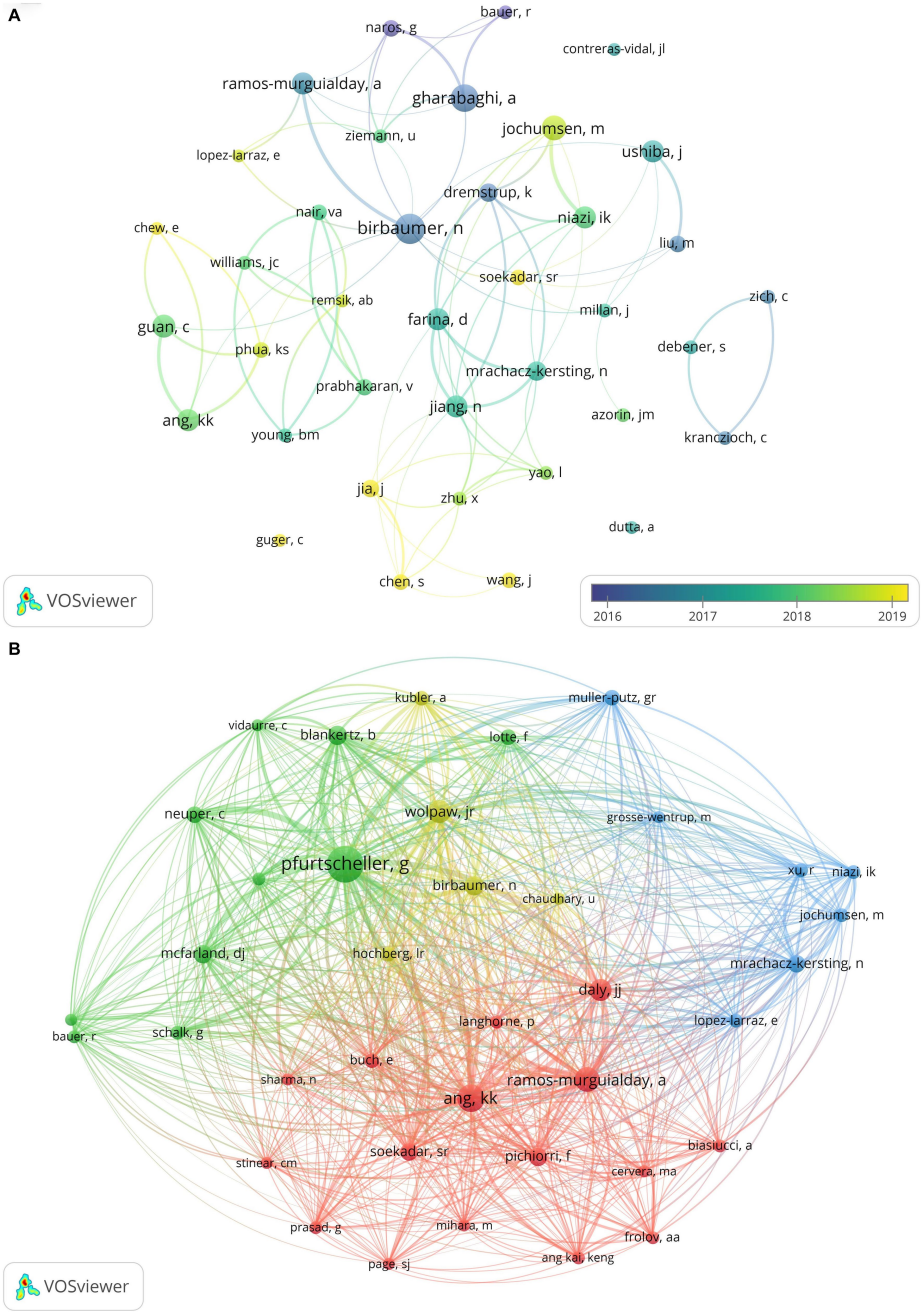
**(A)** Temporal map of author collaboration network. **(B)** Author co-citation network knowledge map.

According to analysis, a total of 2,556 researchers have conducted relevant studies in the field, and the cooperation network time overlay graph of authors who have published eight or more papers is shown in Figure 4A. Birbaumer N (31, 4.47%) from the University of Tubingen in Germany is the most productive author, followed by Gharabaghi A (27, 3.90%) also from the University of Tubingen in Germany, and Jochumsen M (22, 3.18%) from Aalborg University in Denmark. Birbaumer N is among the top in TC, ACPP, and H-index, while Farina D from Imperial College London in the UK has the highest TLS, as shown in Table 3. Figure 4B illustrates the co-citation relationships among the authors, wherein the size of each node corresponds to its frequency of being cited. The co-citation relationships and their strengths are indicated by the connections and thickness between nodes, respectively, while different colors denote different clusters (Chen et al., 2023). Pfurtscheller G (706) from Graz University of Technology in Austria has the highest citation frequency, followed by Ang KK (415) from Nanyang Technological University in Singapore and Ramos-Murguialday A (337) from the University of Tubingen in Germany. These three authors are also among the top three in TLS ranking, as detailed in Table 4.

**Table 3:** Top 10 authors ranked by number of publications.

Rank	Author	Counts (%)	TC	ACPP	H-index	TLS	Location
1	Birbaumer N	31 (4.47%)	2,318	74.77	21	38	Germany
2	Gharabaghi A	27 (3.90%)	904	33.48	20	31	Germany
3	Jochumsen M	22 (3.18%)	398	18.09	11	33	Denmark
4	Jiang N	19 (2.74%)	683	35.95	13	53	Canada
5	Niazi IK	19 (2.74%)	522	27.47	11	37	Denmark
6	Ramos-Murguialday A	19 (2.74%)	1,395	73.42	15	26	Germany
7	Ang KK	18 (2.60%)	1,016	56.44	12	36	Singapore
8	Farina D	18 (2.60%)	685	38.06	13	55	UK
9	Guan CT	18 (2.60%)	950	52.78	12	36	Singapore
10	Ushiba J	18 (2.60%)	663	36.83	11	14	Japan

**Table 4:** Top 10 co-cited authors in citations.

Rank	Co-cited author	Citations	TLS	Location
1	Pfurtscheller G	706	7,613	Austria
2	Ang KK	415	4,654	Singapore
3	Ramos-Murguialday A	337	3,969	Germany
4	Wolpaw JR	290	3,440	USA
5	Daly JJ	269	3,095	USA
6	Blankertz B	216	2,649	Germany
7	Birbaumer N	202	2,478	Germany
8	McFarland DJ	188	2,428	USA
9	Pichiorri F	186	2,377	USA
10	Mrachacz-Kersting N	175	2,285	Germany
11	Soekadar SR	175	1,990	Germany

### Analysis of journals

**Figure 5:**
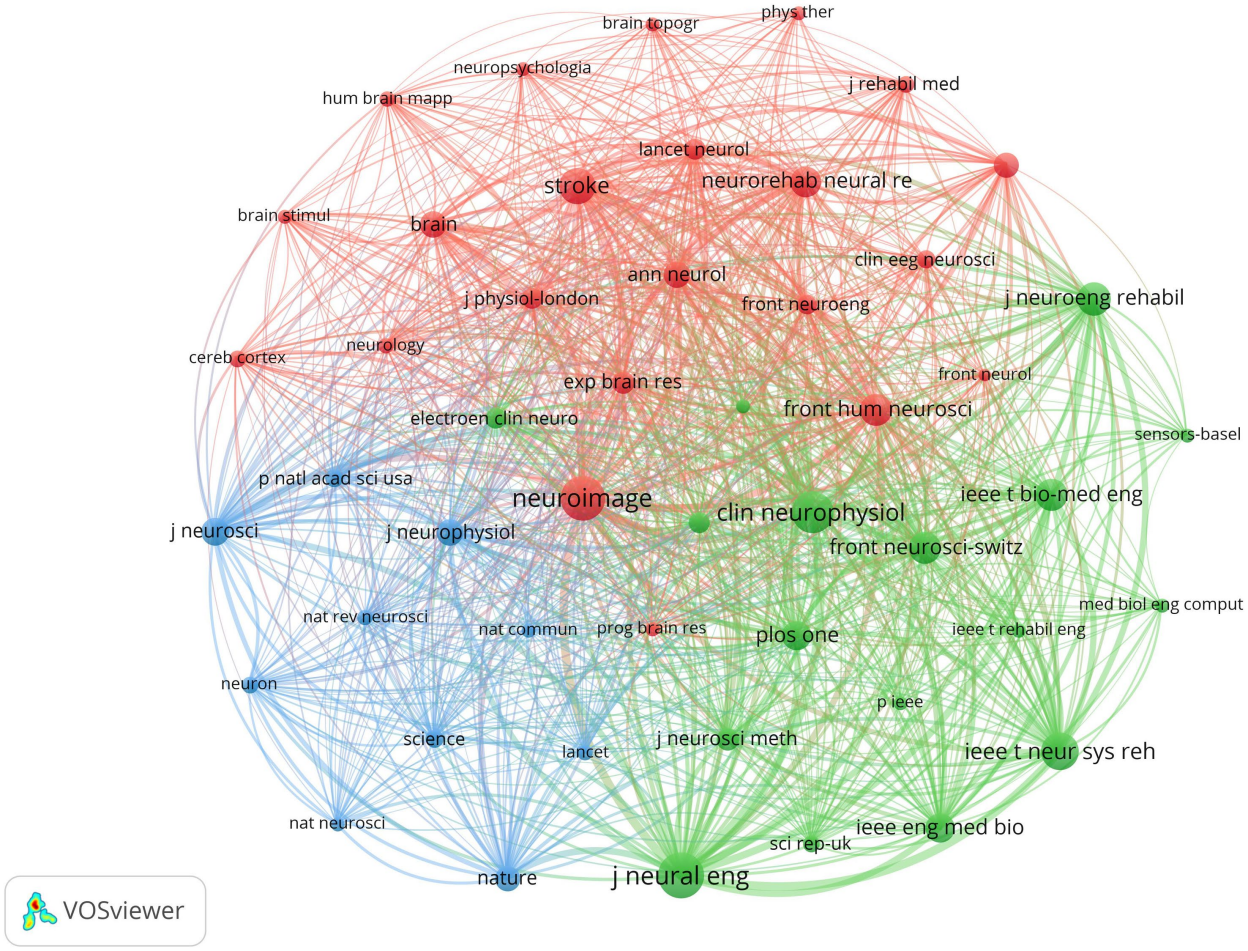
Journal co-citation network knowledge map.

**Figure 6:**
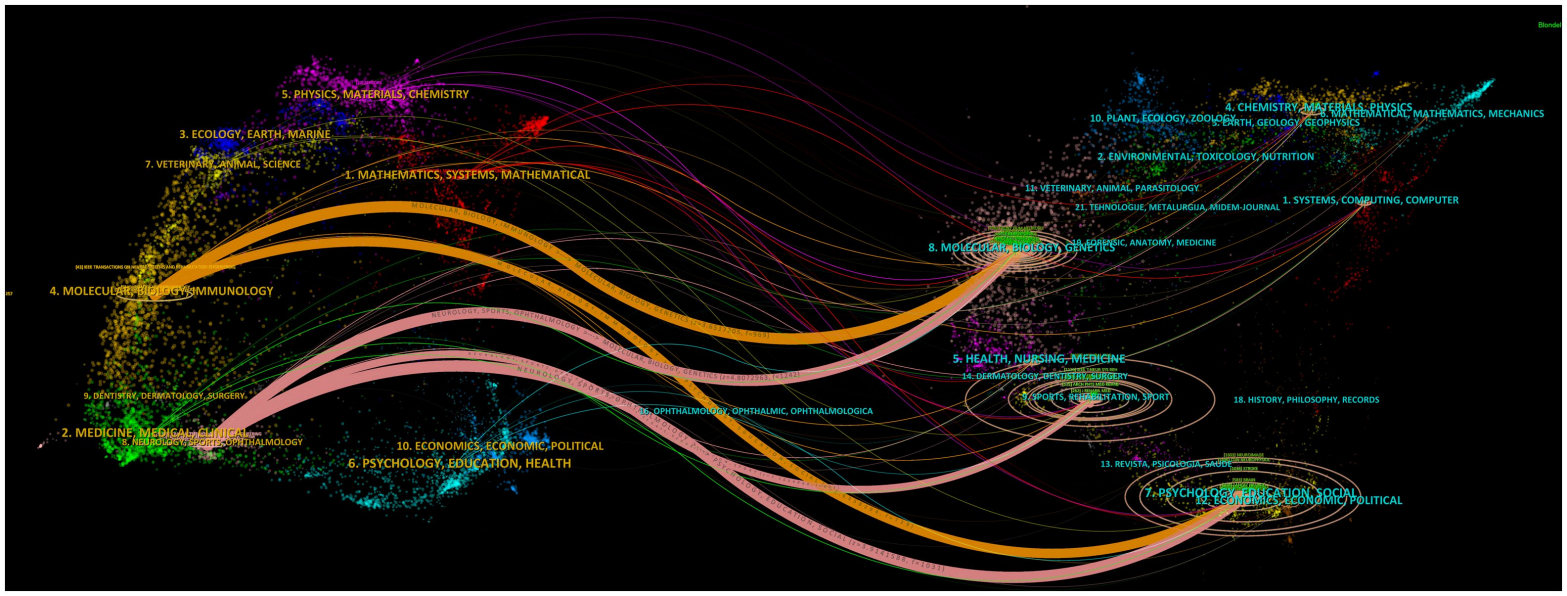
The journal dual-map overlay graph.

The relevant studies included in this paper have been published in 185 journals. Among them, *Frontiers in Neuroscience* (59, 8.51%) has the most publications, followed by *Frontiers in Human Neuroscience* (51, 7.36%) and *Journal of Neural Engineering* (51, 7.36%). *Journal of Neural Engineering* has the highest TC, while *Neuroimage* ranks first in terms of ACPP, H-index, and impact factor (IF), indicating its high-quality and authoritative status in the field. Moreover, all 10 of these journals are classified as Q1 or Q2, signifying a considerable standard of research in the relevant field. The fact that these 10 journals originate from Europe and the United States suggests that they have played a crucial role in advancing scholarship in this domain, as meticulously outlined in Table 5. Figure 5 illustrates the co-citation relationships among journals. Among them, *Journal of Neural Engineering* (1,693) has the highest number of citations, followed by *Neuroimage* (1,605) and *Clinical Neurophysiology* (1,407). The TLS of these three journals also rank in the top three, with *Neuroimage* and *Stroke* being the journals with the highest H-index and IF, respectively. Both of them are Q1 journals, indicating that they are high-quality journals with a high level of academic influence, as shown in Table 6. Figure 6 depicts the citation relationships among journals, where journals on the right are cited by those on the left (Li Y. et al., 2023; Tang et al., 2023; Wang et al., 2023b). Five main citation paths were identified, indicating that papers published in molecular/biology/genetics, sports/rehabilitation/sport, and psychology/education/social journals are mainly cited by those from molecular/biology/immunology and neurology/sports/ophthalmology journals.

**Table 5:** Top 10 journals in terms of the number of published papers.

Rank	Journal	Counts (%)	TC	ACPP	H-index	IF (2021)	Quartile in category
1	FRONT NEUROSCI (Switzerland)	59 (8.51%)	1,153	19.54	71	5.152	Q2
2	FRONT HUM NEUROSCI (Switzerland)	51 (7.36%)	1,216	23.84	87	3.473	Q2
3	J NEURAL ENG (England)	51 (7.36%)	1,709	33.51	89	5.043	Q2
4	IEEE T NEUR SYS REH (United States)	43 (6.21%)	854	19.86	121	4.528	Q1
5	J NEUROENG REHABIL (England)	30 (4.33%)	962	32.07	75	5.208	Q1
6	SENSORS (Switzerland)	22 (3.18%)	414	18.82	132	3.847	Q2
7	FRONT NEUROL (Switzerland)	16 (2.31%)	239	14.94	49	4.086	Q2
8	PLOS ONE (United States)	16 (2.31%)	445	27.81	268	3.752	Q2
9	IEEE T BIO-MED ENG (United States)	15 (2.17%)	447	29.80	172	4.756	Q2
10	NEUROIMAGE (United States)	14 (2.02%)	562	40.14	320	7.400	Q1

**Table 6:** Top 10 co-cited journals in citations.

Rank	Co-cited Journal	Citations	TLS	H-index	IF (2021)	Quartile in category
1	J NEURAL ENG (England)	1,693	69,088	89	5.043	Q2
2	NEUROIMAGE (United States)	1,605	62,258	320	7.400	Q1
3	CLIN NEUROPHYSIOL (Ireland)	1,407	53,086	164	4.861	Q2
4	IEEE T NEUR SYS REH (United States)	1,110	40,638	121	4.528	Q1
5	STROKE (United States)	1,039	41,530	292	10.170	Q1
6	J NEUROENG REHABIL (England)	875	36,037	75	5.208	Q1
7	FRONT HUM NEUROSCI (Switzerland)	838	35,602	87	3.473	Q2
8	IEEE T BIO-MED ENG (United States)	827	30,221	172	4.756	Q2
9	FRONT NEUROSCI (Switzerland)	809	33,791	71	5.152	Q2
10	NEUROREHAB NEURAL RE (United States)	756	33,834	92	4.895	Q1

### Analysis of references

**Figure 7:**
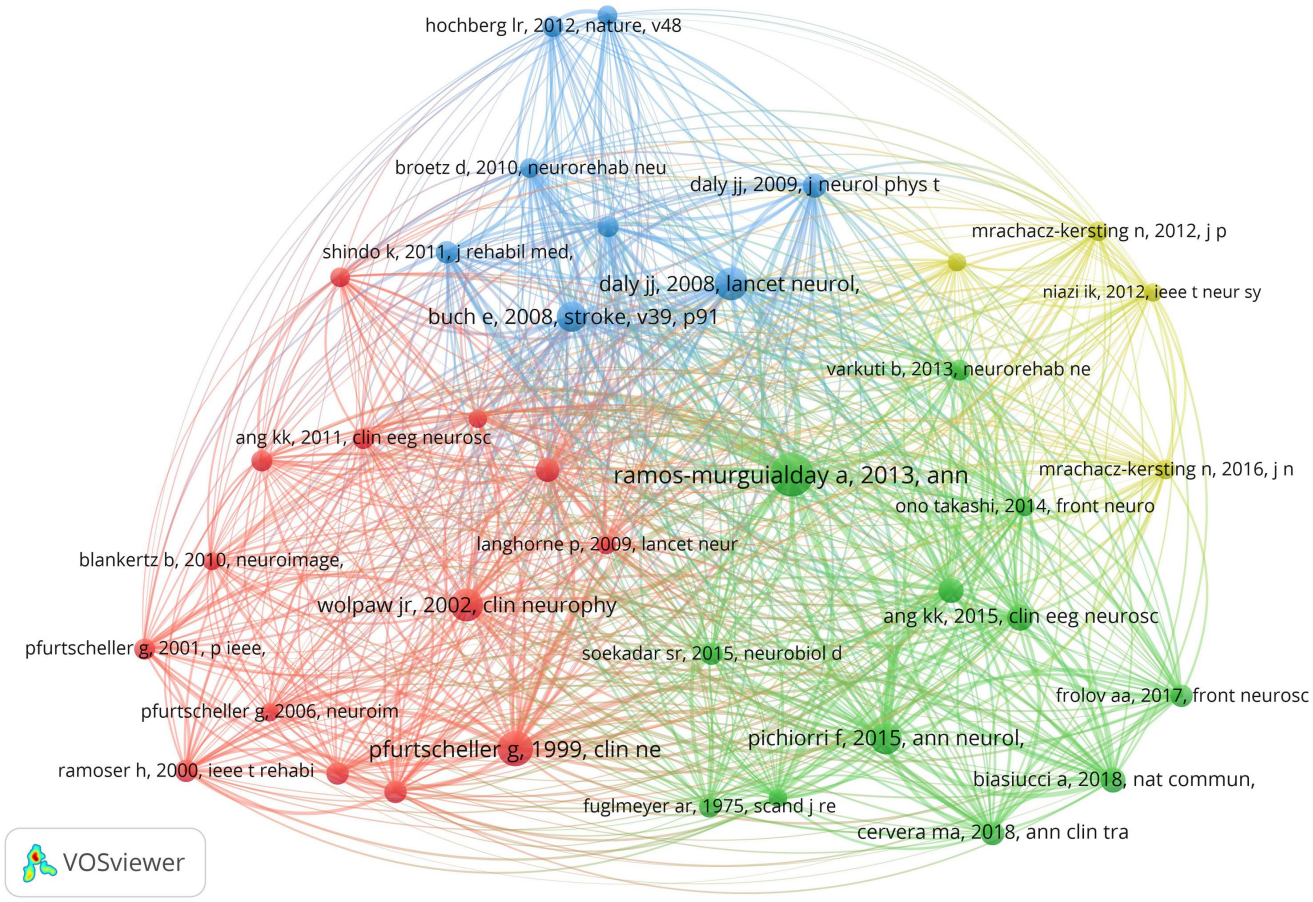
Literature co-citation network knowledge map.

**Figure 8:**
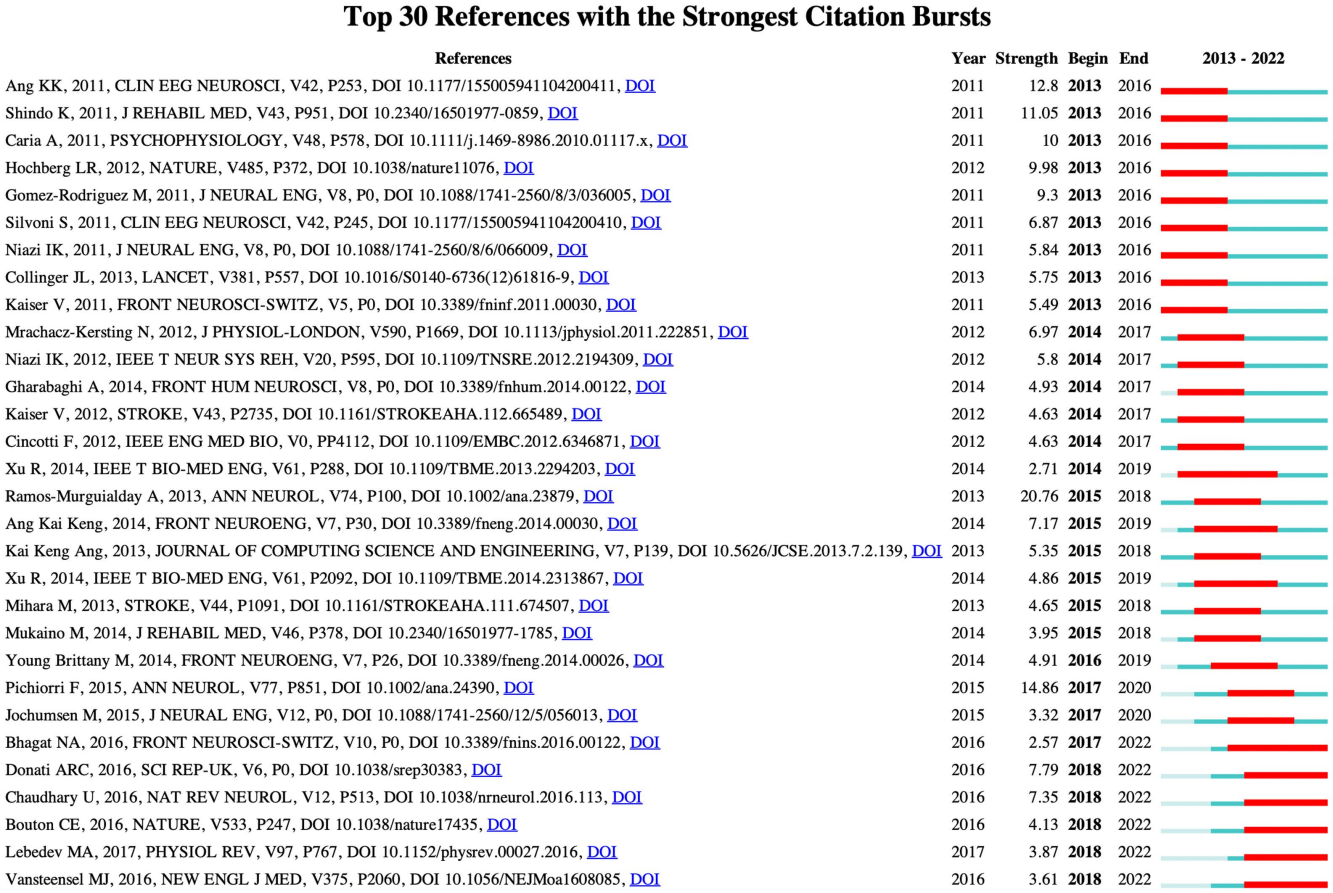
The top 30 references with the highest burst strength.

Figure 7 presents the co-citation networks formed by the publications cited at least 50 times, which intuitively reflects the co-citation relationships among papers. Meanwhile, Table 7 provides the relevant information of the top 10 most frequently cited publications, indicating to some extent the main research content in this field. Document bursts refer to those publications with sudden increases in citation frequency within a brief span of time, and analyzing these publications can provide valuable insights into recent research hotspots and emerging trends within a particular field (Liu Z. et al., 2023; Wang Y. et al., 2023). In this study, we set the duration of document burst to 4 years and filtered out 30 references with high strength of burst, as shown in Figure 8. In the figure, the “Strength” value is indicative of the magnitude of the burst, while “Begin” and “End” respectively indicate the start and end time of the burst, with the blue line representing the time interval and the red line representing the duration of the burst (Xiong et al., 2022; Zhang Y. et al., 2022; Wang et al., 2023a).

**Table 7:** Top 10 co-cited references in citations.

Rank	Co-cited reference	Author and publication year	Citations	TLS	Journal IF (2021)	H-index	Quartile in category
1	Brain-machine interface in chronic stroke rehabilitation: a controlled study	Ramos-Murguialday A, 2013	242	1,605	ANN NEUROL (IF: 11.274)	273	Q1
2	Event-related EEG/MEG synchronization and desynchronization: basic principles	Pfurtscheller G, 1999	166	979	CLIN NEUROPHYSIOL (IF: 4.861)	164	Q2
3	Brain-computer interfaces in neurological rehabilitation	Daly JJ, 2008	148	941	LANCET NEUROL (IF: 59.935)	259	Q1
4	Brain-computer interfaces for communication and control	Wolpaw JR, 2002	146	769	CLIN NEUROPHYSIOL (IF: 4.861)	164	Q2
5	Brain-computer interface boosts motor imagery practice during stroke recovery	Pichiorri F, 2015	135	940	ANN NEUROL (IF: 11.274)	273	Q2
6	Think to move: a neuromagnetic brain-computer interface (BCI) system for chronic stroke	Buch E, 2008	126	941	STROKE (IF: 10.170)	292	Q1
7	A Randomized Controlled Trial of EEG-Based Motor Imagery Brain-Computer Interface Robotic Rehabilitation for Stroke	Ang KK, 2015	101	760	CLIN EEG NEUROSCI (IF: 2.046)	47	Q4
8	Brain-actuated functional electrical stimulation elicits lasting arm motor recovery after stroke	Biasiucci A, 2018	94	632	NAT COMMUN (IF: 17.694)	248	Q1
9	Applying a brain-computer interface to support motor imagery practice in people with stroke for upper limb recovery: a feasibility study	Prasad G, 2010	85	681	J NEUROENG REHABIL (IF: 5.208)	75	Q1
10	Feasibility of a new application of noninvasive Brain Computer Interface (BCI): a case study of training for recovery of volitional motor control after stroke	Daly JJ, 2009	84	649	J NEUROL PHYS THER (IF: 4.655)	44	Q1

### Analysis of keywords

**Figure 9:**
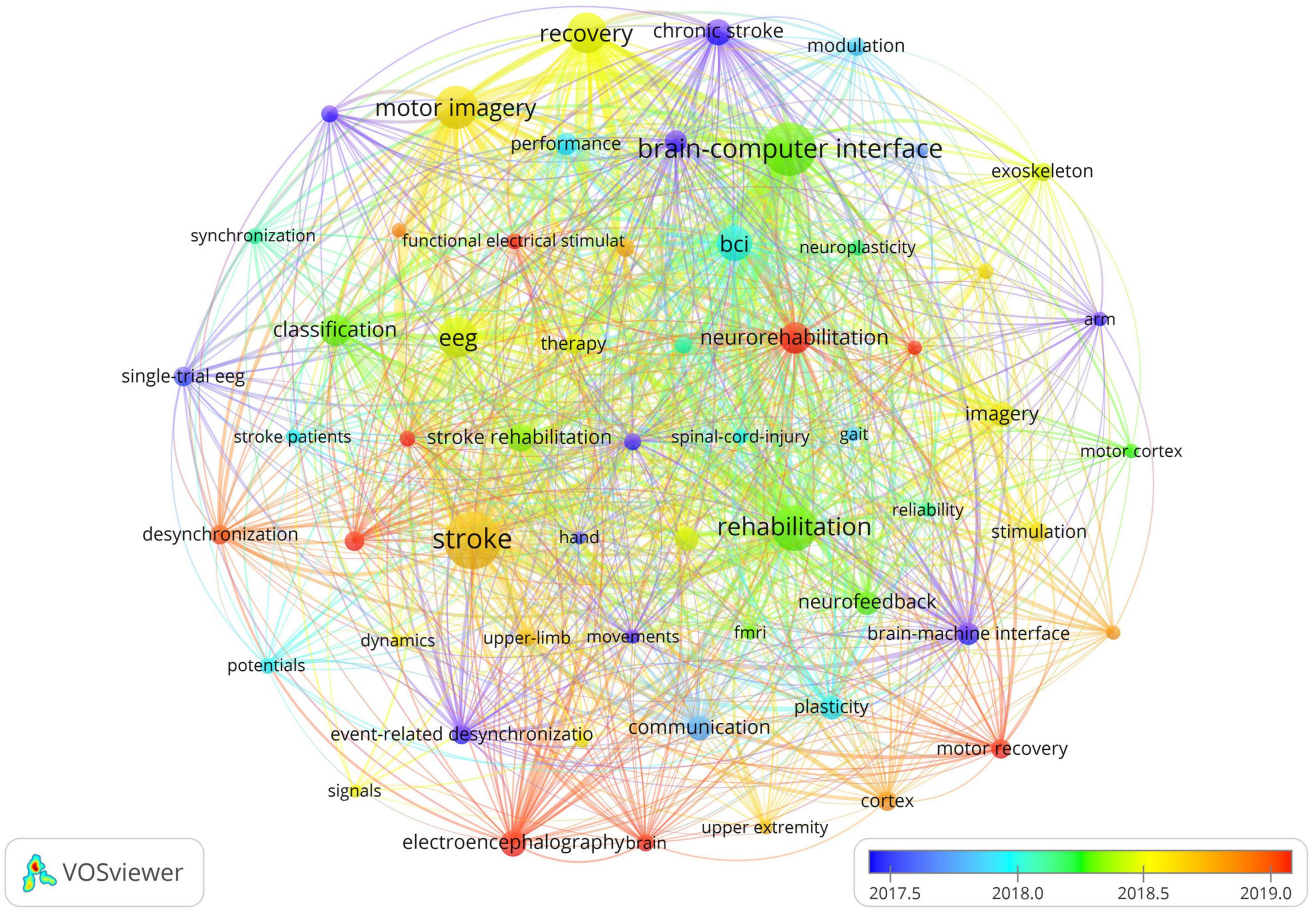
Keyword co-occurrence network knowledge map.

**Figure 10:**
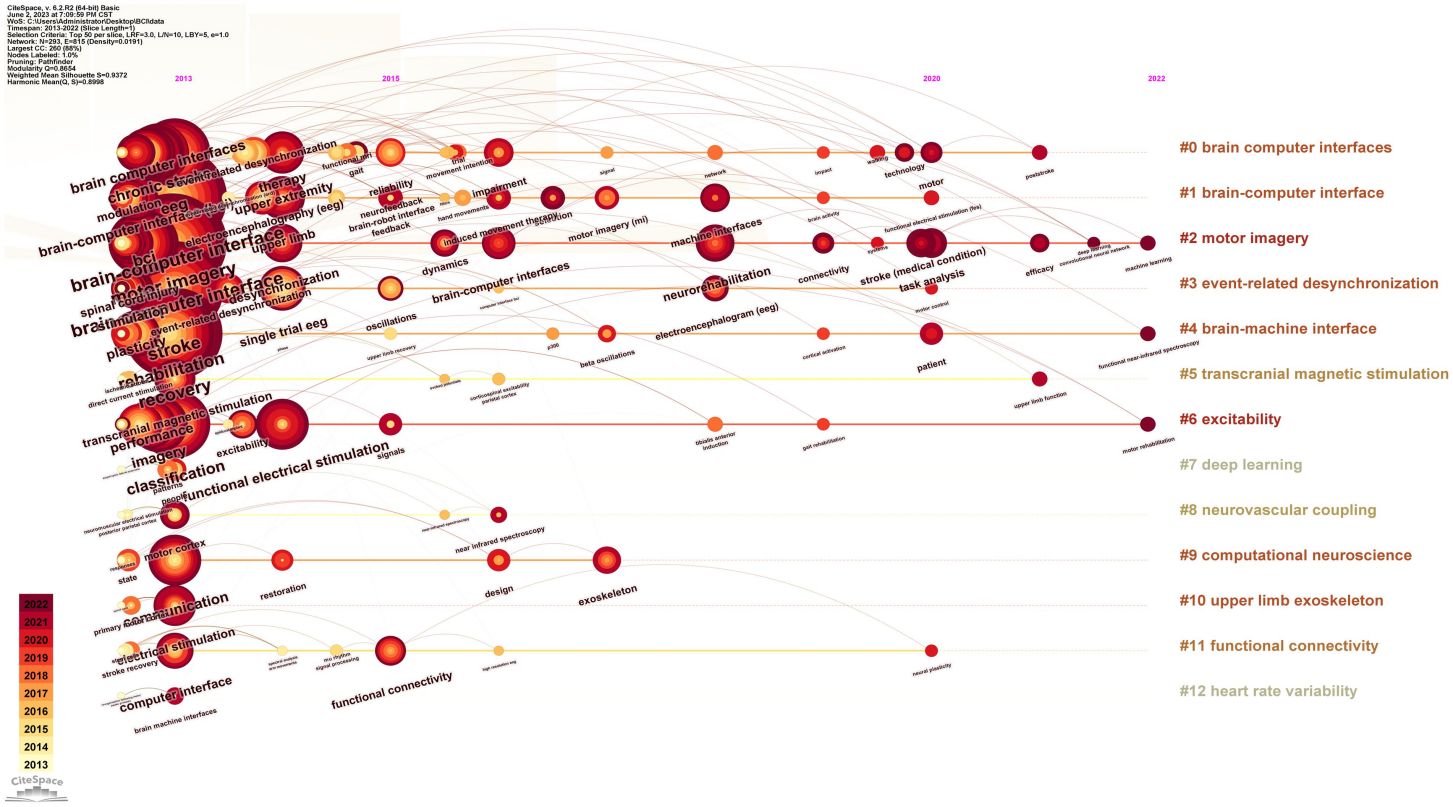
Keyword timeline map.

**Figure 11:**
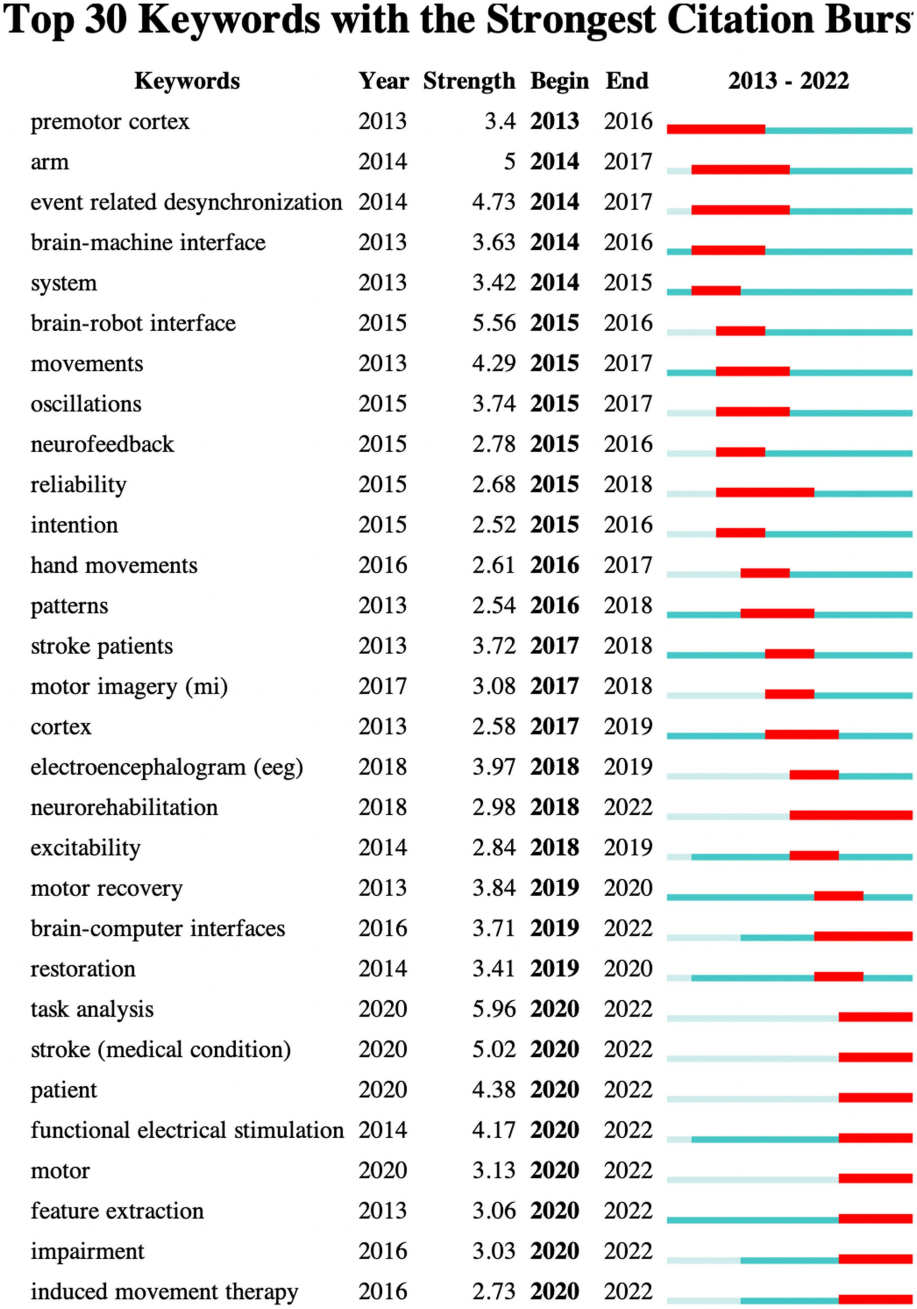
Top 30 keywords with the highest burst strength.

Keywords are succinct summary of the research content of a paper, and analyzing high-frequency keywords can reveal the main research topics in a given field (Xu et al., 2022; Yang J. et al., 2023; Zhang G. et al., 2023). Figure 9 presents the co-occurrence relationships between keywords that appeared no less than 20 times, where the color of the nodes leaning toward warmer shades of red indicates the later average appearance time of the corresponding keyword. Additionally, Table 8 lists the top 40 keywords with high frequency and their TLS, providing insights into the primary research content and hot topics within the field. Thirteen representative cluster labels were formed by clustering the keywords, which were then displayed as a timeline chart in Figure 10, facilitating an intuitive understanding of the evolving trends of each cluster label over time (Lin et al., 2021; Xiao et al., 2023). Furthermore, by setting the duration of burst to 2 years, the top 30 keywords with the highest burst intensity were selected and presented in Figure 11. Through the analysis of burst words, research hotspots and future development trends in the field can be explored, with crucial reference value and guidance significance, particularly when analyzing keywords with sustained burst time extending to the present (Li et al., 2022; Ling et al., 2023).

**Table 8:** Top 40 keywords with frequency.

Rank	Keyword	Frequency	TLS	Rank	Keyword	Frequency	TLS
1	Stroke	338	1,855	21	Therapy	43	270
2	Brain-computer interface	294	1,570	22	Cortex	43	222
3	Rehabilitation	217	1,328	23	Event-related desynchronization	42	290
4	Motor imagery	188	1,142	24	Stimulation	41	267
5	Recovery	174	1,167	25	Motor recovery	40	243
6	EEG	171	938	26	Single-trial EEG	40	243
7	BCI	130	787	27	Functional electrical-stimulation	40	239
8	Neurorehabilitation	105	636	28	Exoskeleton	38	240
9	Classification	102	565	29	Modulation	36	241
10	Stroke rehabilitation	80	453	30	Transcranial magnetic stimulation	35	167
11	Chronic stroke	75	440	31	Mental practice	33	211
12	Electroencephalography	70	396	32	Reorganization	31	212
13	Imagery	68	433	33	Activation	31	194
14	Communication	66	387	34	Upper-limb	31	194
15	Movement	61	356	35	neuroplasticity	30	188
16	Neurofeedback	59	406	36	Synchronization	28	188
17	Plasticity	58	366	37	Functional connectivity	28	166
18	Brain-machine interface	55	342	38	Potentials	26	153
19	System	54	345	39	Stroke patients	25	143
20	Performance	53	335	40	Motor cortex	25	120

## Discussion

This study employs bibliometric methods to comprehensively and systematically examine the relevant literature on BCI technology in stroke research over the past decade. Through visualizing knowledge maps, the study presents an intuitive representation of the knowledge structure and developmental trajectory in this field from multiple perspectives. The findings indicate that BCI technology has received extensive attention and attracted increasing numbers of researchers in the field of stroke research, resulting in a prolific output of research achievements and promoting the vigorous development of this field.

As one of the leading research nations, countries such as the United States, Germany, the United Kingdom, and Spain have a relatively early average publication year for academic publications. During the early stages of research in this field, they made outstanding contributions, laying a solid foundation of knowledge for subsequent studies. In comparison, China, as the top-producing country, had relatively fewer relevant research achievements prior to 2018 and was in its initial stages of development. However, in recent years, due to strong support from national policies and funding, China has embarked on the fast lane of development in this area, producing a large number of academic research achievements, and has leapt to become the top-performing country in terms of both total publications and annual publications. Nevertheless, the ACPP of these research results ranks lowest among the top 10 countries/regions in terms of publication output, indicating the overall low research quality of Chinese publications and a failure to achieve widespread dissemination in this field. Hence, the insight gained is that striving for academic prestige and influence in a research field should not only prioritize the quantity of research achievements, but also pay more attention to their quality. Therefore, in the future development of this field, China should not only guarantee the quantity of research achievements, but also strive to improve the level of scientific research and increase the output of high-quality academic achievements in order to enhance its international influence. Germany has also made outstanding contributions to this field, with the highest TLS, indicating that it has established extensive cooperation relationships with other countries/regions, particularly the United States, Spain, the United Kingdom, Italy, Switzerland, and Denmark. Switzerland, while ranking only 10th in terms of publication output, has the highest ACPP, indicating a high research level, with academic achievements enjoying widespread recognition and citation from many scholars, thereby demonstrating a high academic influence. A highly cited review study on BCI technology and its applications in severe stroke and spinal cord injury motor rehabilitation, co-authored with other countries, was published by Switzerland (Chaudhary et al., 2016). Furthermore, it is worth noting that 80% of the top 10 countries/regions with the highest publication output are located in North America and Europe, indicating that they are the main strongholds of this research field.

The University of Tubingen in Germany has emerged as a leading institution in the field, with a notable abundance of publications. These publications have contributed significantly to early-stage research in the field, with an average time of publication being relatively early. One of their highly cited papers evaluated the efficacy of BCI training in the rehabilitation of chronic stroke, revealing that BCI training can significantly improve upper limb Fugl-Meyer assessment motor scores in 32 individuals suffering from chronic stroke who were randomly assigned to groups. Notably, this improvement in motor scores was found to be correlated with changes in laterality index of functional magnetic resonance imaging and electromyographic activity of the paretic hand, thus indicating the potential of BCI training to induce motor function improvement in chronic stroke patients without residual finger movements (Ramos-Murguialday et al., 2013). Aalborg University in Denmark not only ranks high in terms of publication output but also boasts the highest TLS, indicating its highly frequent collaborations with other institutions, particularly with the New Zealand Coll Chiropract, University of Waterloo, Imperial College London, University of Gottingen, Auckland University of Technology, and Shanghai Jiao Tong University. Among their highly cited works is a study that evaluated the effects and potential mechanisms of BCI training based on Hebbian associative principles in 22 individuals with chronic stroke using a double-blind sham-controlled design. Results revealed that precise coupling between brain commands and afferent signals is essential for the reported behavioral, clinical, and neurophysiological changes, which may become a driving principle for future BCI rehabilitation design (Mrachacz-Kersting et al., 2016). Although Tecnalia in Spain has relatively fewer publications, it ranks first in the ACPP ranking, indicating its high-quality academic achievements that have gained widespread recognition and citations from scholars, thus possessing a high level of academic influence in the field. The institution recently conducted a study examining neural plasticity in the motor network of severely impaired chronic stroke patients following a therapy based on electroencephalogram (EEG)-BCI. The results demonstrated that BCI contributes to the enhancement of the ipsilesional brain activity and proprioceptive function of the affected hand, thereby leading to a reorganization of both contralesional and ipsilesional somatosensory and motor assemblies, as well as afferent and efferent connection-related motor circuits. This reorganization facilitates the partial restoration of the original neurophysiology of the motor system, even in cases of severe chronic stroke (Caria et al., 2020). Despite ranking third in terms of research paper output, Fudan University in China shows a relatively low ACPP, indicating the need for further improvement in its research level. To address this, it is crucial to enhance collaboration and communication with domestic and international research institutions. Additionally, attention should be paid to the fact that Fudan University’s publications have a relatively late average publication year, primarily focusing on recent research areas.

Birbaumer N, from the University of Tubingen in Germany, is not only the most prolific author in the field but also one of the top 10 most cited authors. Additionally, his TC, ACPP, and H-index are also among the highest, indicating his significant academic influence and important contributions to the field. One of his highly cited studies conducted a meta-analysis and assessed the effectiveness of BCI in post-stroke motor rehabilitation, revealing that BCI induces functional and structural neural plasticity at a subclinical level (Cervera et al., 2018). Farina D, from Imperial College London in the UK, ranks first in TLS, which indicates close collaboration and communication with other scholars, particularly Jiang N, Dremstrup K, Mrachacz-Kersting N, Niazi IK, and Jochumsen M. Pfurtscheller G, from Graz University of Technology in Austria, has the highest citation frequency of any author and his academic achievements have been widely cited and disseminated. One of his highly cited studies reviewed the current state of BCI technology and the challenges to overcome for its future development (Wolpaw et al., 2002). It is worth noting that high productivity and high citation authors mostly come from North America and Europe, especially Germany and the United States. In contrast, there has not been a prominent research team with significant academic influence in the field among Chinese researchers, and there is room for improvement in scientific research strength and level, which requires continuous efforts to achieve better breakthroughs.

*Frontiers in Neuroscience* is one of the top 10 journals in the field in terms of the number of publications and citation frequency. One of its highly cited articles reported a clinical trial that utilized BCI training on 74 stroke patients suffering from severe upper limb paralysis, which demonstrated that incorporating BCI control into exoskeleton-assisted physical therapy can improve motor function in stroke patients with varying degrees of severity, affected regions, and duration since onset (Frolov et al., 2017). *Journal of Neural Engineering* is the top-ranked journal in terms of citation frequency and TLS, and is among the top three journals with respect to publications. Furthermore, it has established strong co-citation relationships with other journals such as *Neuroimage*, *Clinical Neurophysiology*, *Frontiers in Neuroscience*, *Nature*, *Lancet*, *Science*, *Plos One*, *IEEE Transactions on Neural Systems and Rehabilitation Engineering*, *Stroke*, *Journal of NeuroEngineering and Rehabilitation*, *Frontiers in Human Neuroscience*, and *Journal of Neuroscience*. One of its high-profile papers evaluated the advantages and limitations of EEG-based BCI paradigms from various perspectives and analyzed potential issues of EEG-based BCI systems, proposing possible solutions (Abiri et al., 2019). Furthermore, *Neuroimage* is the Q1 journal with the highest ACPP and h-index, while *Stroke* is the high-cited Q1 journal with the highest IF. This indicates that these two journals have high recognition and authority, and their published articles are of great academic reference value. When publishing research in this field, submitting to these highly productive journals can be a priority, and when searching for relevant literature, these highly cited journals’ collected papers can be the first choice.

Highly cited literature typically reflects high-quality research and academic impact while also providing insights into the main research focus in a given field (Wu et al., 2021; Zhu et al., 2021; Li R. et al., 2023). Therefore, analyzing highly cited literature can offer preliminary understanding of the research trends and direction in that field. A highly cited study by Pfurtscheller and Lopes Da Silva (1999) introduced the basic principles of event-related synchronization and desynchronization in EEG and magnetoencephalogram (MEG). The study found that the neural structure of the brain exhibits different responses to event-related potential (ERP) and event-related desynchronization (ERD) or synchronization (ERS), with ERD/ERS being highly specific frequency band. Daly and Wolpaw (2008) conducted a study on the application of BCI for improving motor control in patients after stroke. The technology utilizes EEG brain signals to indicate the current state of brain activity to patients and guides them to modulate abnormal brain activity, thereby facilitating the development of activity-dependent brain plasticity. Pichiorri et al. (2015) conducted a randomized controlled study on 28 patients with severe functional impairment following subacute stroke to evaluate the efficacy of motor imagery (MI) monitored by BCI on post-stroke motor recovery. The results demonstrated that the BCI group achieved better functional recovery outcomes compared to the control group, including a significantly increased probability of clinical-relevant Fugl-Meyer score improvement. This indicates that incorporating BCI technology can assist in MI practice, demonstrating the potential of MI in rehabilitation, and improving the severe motor impairment in subacute stroke patients. Buch et al. (2008) conducted a study on a BCI system based on MEG with 8 chronic stroke patients suffering from hand paralysis. The results indicated that these patients were able to achieve volitional control of the neuro-magnetic activity features recorded from the central region of the scalp after BCI training, and control gripping movements using a mechanical hand orthosis. Ang et al. (2015) investigated the effectiveness of an EEG-based MI BCI system combined with MIT-Manus shoulder-elbow robotic feedback (BCI-Manus) in chronic stroke patients with upper limb hemiparesis. The results demonstrated that the BCI-Manus therapy is effective and safe for the rehabilitation of severe upper limb hemiparesis in post-stroke patients, and the revised brain symmetry index (rBSI) could serve as an important prognostic indicator for BCI-based stroke rehabilitation. The study by Biasiucci et al. (2018) found that the combination of BCI with functional electrical stimulation (FES) is more effective in promoting motor recovery of chronic stroke survivors than sham FES. This recovery is significant, clinically relevant, and durable, and is associated with quantitative features of functional neuroplasticity. Prasad et al. (2010) investigated the role of a BCI system in providing computer game-based neurofeedback during the MI phase of a protocol to 5 chronic stroke hemiparetic patients. The results showed that BCI-supported MI is a feasible intervention that can be combined with physical practice and MI practice of rehabilitation tasks as part of post-stroke rehabilitation programs. Daly et al. (2009) demonstrated the feasibility of combining BCI with FES training for post-stroke motor learning, achieving highly accurate control of brain signals that were reliably used to trigger FES devices for isolated index finger extension. Analyzing burst literature, especially those whose burst has continued until recently, can explore research hotspots and future development trends in the field to some extent. It is noteworthy that six of the papers had burst times extending to and after 2022, indicating that the research findings of these papers may be more closely related to the latest scientific frontiers and have important reference value for understanding the future development trends in the field. Bhagat et al. (2016) demonstrated the feasibility of detecting motor intentions from the brain activity of chronic stroke patients using an asynchronous EEG-based BCI technology. By measuring the movement-related cortical potentials with an optimized EEG electrode placement, motor intentions can be accurately inferred. Successful intention detection can trigger upper-limb exoskeleton movements and guide the movements with real-time sensory feedback, encouraging active participation of the patients. Donati et al. (2016) implemented a multi-stage BCI-based gait neurorehabilitation protocol to train eight chronic spinal cord injury (SCI) paraplegic patients with the aim of restoring their motor function over a period of one year. The results showed that all patients exhibited neurological improvements in various somatosensory domains such as pain localization, fine/rough touch, and proprioception across multiple dermatomes. Utilizing electrocardiogram measurements, patients also regenerated autonomous motor control in crucial muscles below the level of SCI, leading to significant improvements in their walking index. Bouton et al. (2016) successfully restored motor function in a paralyzed human by using intracortically recorded signals to control muscle activation for the first time. Lebedev and Nicolelis (2017) conducted a comprehensive review of the research progress in BCI. The classic objectives of BCI are to reveal and exploit the operating principles and plasticity characteristics of distributed and dynamic brain circuits, and to restore the mobility and sensation of severely disabled patients through the creation of novel therapies. In recent years, BCI research has also introduced new neurorehabilitation strategies, providing new insights for clinical applications. Vansteensel et al. (2016) described a communication method for patients with advanced amyotrophic lateral sclerosis, utilizing a fully implanted BCI consisting of dura-subpial electrodes placed on the motor cortex and an emitter placed subcutaneously on the left side of the chest. After 28 weeks of electrode placement, patients were able to accurately and independently control a computer typing program at a rate of approximately two letters per minute by attempting to move the hand contralateral to the implanted electrodes. The analysis of the highly cited and burst literature presented above can aid in acquiring a foundational knowledge of the field, identifying research hotspots, and predicting future developmental trends. These high-quality publications possess significant academic impact and have played a crucial role in driving the progress of the field. Through further examination of these publications, we can gain a better understanding of the current state and future directions of the field, thereby enhancing our research capabilities and the quality of our research outcomes.

The co-occurrence analysis of keywords demonstrates that stroke, BCI, rehabilitation, MI, recovery, EEG, neurorehabilitation, and classification are the major research topics in this field. By clustering these keywords and displaying them in a timeline graph, it can be observed that the clustering labels of #2 MI, #4 BCI, and #6 excitability have continued to evolve to date, which to some extent reflects the current research hotspots. Burst keywords refer to the research hotspots that have been frequently studied during a certain period of time. In recent years, burst keywords in this field include neurorehabilitation, BCI, task analysis, stroke patient, FES, feature extraction, motor impairment, and induced movement therapy. These research hotspots not only reflect the current development trends but also provide valuable insights for future research. In the field of neurorehabilitation, research on BCI, stroke patient rehabilitation technology, and motor function recovery has become major research hotspots. In the future, further in-depth research could explore issues such as task analysis, FES, and feature extraction, aiming to improve the effectiveness of rehabilitation treatments and provide beneficial support for stroke rehabilitation practice.

## Strengths and limitations

This study employs bibliometric analysis and visualization techniques to comprehensively and systematically analyze the research on BCI technology in the field of stroke for the first time. From multiple perspectives, including collaboration networks, co-citation networks, and co-occurrence networks, the knowledge framework and development trajectory of this research field are presented. Moreover, the study explores research hotspots and future development trends, providing valuable references and directions for further research. However, there are certain limitations to this study. Firstly, the literature data was solely obtained from the WoSCC database, potentially leading to the omission of relevant literature from other sources. Nonetheless, it is important to highlight that the WoSCC database is widely recognized as the most commonly employed database for bibliometric research (Zhong and Lin, 2022; Dong et al., 2023; He et al., 2023; Jiang et al., 2023; Li M. et al., 2023; Liu T. et al., 2023). Additionally, some recently published high-quality literature may be underestimated. Finally, the incomplete dataset of this year has unavoidably restricted its inclusion in this study.

## Conclusion

This study encompasses a total of 693 relevant literature sources, published by 2,556 scholars from 975 institutions across 53 countries/regions and collected by 185 journals. Notably, over the past decade, both the annual publications and citations related to BCI technology in stroke research have demonstrated an overall upward trend, indicating rapid development and significant attention in this field. China and the United States stand out as highly productive countries, while the University of Tubingen has made the greatest contributions. Birbaumer N and Pfurtscheller G, respectively, rank as the most prolific author and highest cited author, both of whom have earned high academic recognition and impact through their outstanding work. Regarding journals, *Frontiers in Neuroscience* published the most literature, while *Journal of Neural Engineering* had the highest citation frequency. The research hotspots in this field encompass crucial keywords such as stroke, BCI, rehabilitation, MI, motor recovery, EEG, neurorehabilitation, neural plasticity, task analysis, FES, motor impairment, feature extraction, and induced movement therapy. These hotspots keywords to some extent reflect the development trends and frontier research directions in this field. However, BCI technology still faces numerous challenges in stroke research, including electrode stability, complexity of signal decoding, long-term stability and reliability, adaptability to individual differences, and limitations in clinical applications. Therefore, future research should focus on improving the precision and stability of the technology, enhancing user-friendliness, increasing adaptability to individual differences, and strengthening measures for safety and risk management. Overall, this study, presented in the form of a knowledge graph, comprehensively and systematically showcases the widespread and in-depth literature resources related to BCI technology in the stroke field. This assists scholars in conveniently gaining a more profound understanding of the development and prospects of this field, thereby promoting further research efforts.

## Data availability statement

The original contributions presented in the study are included in the article/supplementary material, further inquiries can be directed to the corresponding author.

## Author contributions

FL, DZ, and ZH designed the study and performed software analysis. FL, JC, KT, XL, and ZH contributed to data collection and verification. FL and ZH drafted the manuscript. DZ, JC, KT, and XL revised and approved the final version of the manuscript. All authors read and approved the submitted version.

## Funding

The study was supported by the Innovation Project of Guangxi Graduate Education (No. YCBXJ2022009), Guilin Science Research and Technology Development Program (No. 2020011208-5), and Self-Funded Research Project of the Health Commission of Guangxi Zhuang Autonomous Region (No. GZZC2019175).

## Conflict of interest

The authors declare that the research was conducted in the absence of any commercial or financial relationships that could be construed as a potential conflict of interest.

## Publisher’s note

All claims expressed in this article are solely those of the authors and do not necessarily represent those of their affiliated organizations, or those of the publisher, the editors and the reviewers. Any product that may be evaluated in this article, or claim that may be made by its manufacturer, is not guaranteed or endorsed by the publisher.

## Footnotes

^1^https://bibliometric.com/
